# Perfluorobutane CEUS for early-stage cervical lymphoma: diagnostic value of the postvascular phase starfield sign

**DOI:** 10.1038/s41598-025-13771-0

**Published:** 2025-08-05

**Authors:** Xia Liang, Xian-Tao Zeng, Zhi-Liang Hong, Xiao-Ying Lin, Miao-jiao Su, Jian-Chuan Yang, Song-Song Wu

**Affiliations:** 1 Shengli Clinical Medical College of Fujian Medical University, Fuzhou, China; 2 Department of Ultrasound, Fujian Provincial Hospital, No.134 Dongjie, Fuzhou, 350001 Fujian China; 3 Fuzhou University Affiliated Provincial Hospital, Fuzhou, China

**Keywords:** Perflubutane, Cervical lymphoma, Contrast-enhanced ultrasound, Postvascular phase, Lymph node, Starfield sign, Cancer, Oncology

## Abstract

Early differentiation of cervical lymphoma from benign lymphadenopathy remains challenging on conventional imaging. This study assesses the diagnostic efficacy of perfluorobutane contrast-enhanced ultrasound (CEUS) in differentiating early-stage cervical lymphoma from benign lymph nodes (LNs). From November 2023 to January 2025 we prospectively enrolled patients suspected of having cervical lymphoma based on ultrasound (US) findings and scheduled for LN biopsy. All patients underwent CEUS to evaluate LN vascular (5–60 s post-injection) and postvascular (10–30 min post-injection) phases before biopsy. Histopathology served as the reference standard. Diagnostic performance metrics, including sensitivity, specificity, and accuracy, were calculated. Logistic regression analyzed the area under the receiver operating characteristic curve (AUC) for US, CEUS, and combined features. Forty-seven LNs (23 lymphomas, 24 benign) were analyzed. The sensitivity of the postvascular phase starfield sign was 91.30%, specificity was 83.33%, positive predictive value was 84.00%, negative predictive value was 90.91%, and the AUC was 0.87 (95% CI 0.76–0.98). The AUC for CEUS was 0.89 (95% CI 0.79–1.00), and the AUC for the combination of postvascular phase and US features was 0.92 (95% CI 0.82–1.00), significantly higher than that for US features alone (AUC, 0.68; 95% CI 0.53–0.84; *P* < 0.05). Perflubutane CEUS can effectively distinguish between cervical lymphoma and benign LNs. The postvascular phase starfield sign demonstrates significant diagnostic efficacy and could improve clinical management strategies.

## Introduction

Lymphoma accounts for 12% of all malignant tumors in the head and neck region, making it a common malignancy^1^. Early diagnosis of lymphoma is crucial for patient prognosis^2,3^. Because most patients with cervical lymphoma have no obvious clinical symptoms in the early stages; sometimes, cervical lymphadenopathy is the only clinical manifestation^4^. Although ultrasound is the preferred imaging modality for superficial tumors^5^, the accuracy of conventional ultrasound (US) in diagnosing cervical lymphoma is not high. Studies have shown that lymphomas share similar imaging characteristics with non-malignant lymph nodes(LNs)^6,7^, mainly because the US features of early lymphomas overlap with those of benign reactive hyperplastic LNs, making differentiation difficult^8–11^. Ultrasound-guided core needle biopsy(CNB) or histological biopsy is invasive, costly, and not the preferred diagnostic method, Although PET-CT has a relatively high diagnostic accuracy for lymphoma, its radiation exposure and high cost make it unsuitable as a first-line diagnostic method. Contrast-enhanced ultrasound (CEUS) uses microbubble backscatter to enhance US imaging performance^12^, CEUS can dynamically assess the microvascular status and blood supply of LNs in real-time^13^. To date, multiple studies have shown that using SonoVue as a contrast agent in CEUS performs better in diagnosing benign cervical lymphadenopathy and lymphoma, with higher accuracy than conventional US^14^, The sensitivity of lymphoma diagnosis is 80.00%, but the specificity is very low, only 47.62%^15^. Therefore, it is necessary to find more accurate, non-invasive, and relatively inexpensive diagnostic methods, The diagnostic value of using perfluorobutane in CEUS to evaluate suspected cervical lymphoma has not been reported.

Perflubutane was initially widely used for diagnosing focal liver nodules because it can be phagocytized by hepatic macrophages and provide prolonged imaging enhancement^16–19^. This phase is defined as the postvascular phase (10–30 min after injection, also known as the Kupffer phase). Similar to the liver, normal LNs contain macrophages in their cortex and medulla^20,21^. Studies have found that SonoVue microbubbles can also be phagocytized by macrophages within LNs for up to four hours^22^. Goldberg et al.^23^. confirmed the presence of perflubutane microbubbles in macrophages within LNs under a scanning electron microscope after subcutaneous injection. Because early-stage lymphoma has a similar blood supply pattern to benign LNs^24^, conventional contrast-enhanced imaging lacks specificity. Therefore, in this study, we hypothesize that the vascular phase and postvascular phase of perflubutane may help improve the early diagnosis of suspected cervical lymphomas. The aim is to explore the diagnostic value of perfluorobutane CEUS in differentiating suspected cervical lymphoma from benign LNs.

## Material and method

### Participants

The Fujian Provincial Hospital review committee approved this prospective, single-center study (Ethics No. K2023-11-006), which adhered to the Declaration of Helsinki and national guidelines. All participants provided written informed consent. From November 2023 to January 2025, we prospectively collected data on patients presenting to Fujian Provincial Hospital, affiliated with Fuzhou University, with suspected cervical lymphoma who were scheduled for ultrasound-guided CNB or surgical biopsy following an US examination.

Inclusion criteria were as follows:

1. Painless, enlarged cervical LNs persisting for more than 3 months;
2. Maximum LN diameter < 4 cm;
3. Indeterminate ultrasound features include cortical thickening, a visible but thinned or partially absent hilum, absence of abnormally rich non-hilum blood flow, no obvious liquefaction, calcification, hyperechoic nodules, or reticular extremely hypoechoic areas.
4. No history of malignancy or tuberculosis.

Exclusion criteria were as follows:

1. Histologically confirmed malignancy with cervical LN metastases or ultrasound features strongly suggestive of metastasis;
2. Tuberculosis;
3. Acute lymphadenitis (local redness, swelling, heat, and pain within < 3 months, accompanied by high fever);
4. Contraindications to CEUS, including severe allergic reactions;
5. Pregnancy or lactation;
6. Severe heart failure, coronary heart disease, or pulmonary hypertension.

**Fig. 1:**
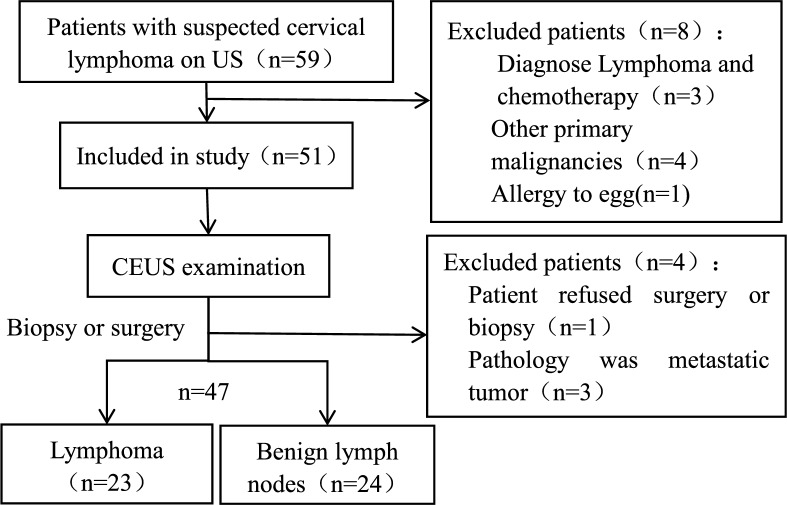
Flowchart of participant selection. CEUS = contrast-enhanced, ultrasoundLN = lymph node.

A total of 47 patients met the inclusion criteria (Fig. 1).

### Equipment and materials

US examinations were conducted using a Philips EPIQ7 color Doppler system with a C12-5 probe (5–12 MHz). The contrast agent used was Sonazoid (Daiichi Sankyo), composed of microbubbles containing perfluorobutane gas. It was prepared by mixing dry powder with 2 mL of 0.9% NaCl.

### US and CEUS examination

Patient data, including age, gender, and lesion type, were recorded prior to imaging. All US and CEUS examinations were conducted by a single experienced radiologist (W.S.S), who has 20 years of experience in superficial US and 15 years in CEUS. Patients were scanned in the supine position with the neck fully exposed. Standard US parameters, such as LN size, shape, margins, internal echoes, cystic changes, homogeneity, hilum status, and vascularity, were assessed.

For CEUS, the US system was set to CEUS mode (mechanical index: 0.20–0.25), and the contrast agent (0.015 mL/kg) was manually injected via the antecubital vein, followed by a 5 mL saline flush. A timer and video recording were started simultaneously at the time of injection. Imaging was performed continuously from 5 to 180 s, with an additional assessment in the postvascular phase (10–30 min post-injection). If the LN assessment was unsatisfactory, a second injection was administered after 15 min. All images and videos were stored for further analysis.

Immediately after CEUS, patients underwent lesion CNB or surgical biopsy to obtain a definitive pathological diagnosis. All participant slides were independently reviewed by a dedicated thyroid pathologist (W.C), who has over 15 years of experience in superficial LN pathology and was blinded to the participants’ clinical histories and US data.

### Image interpretation

US and CEUS images were independently reviewed by two radiologists (L.X and Z.X.T, with > 8 years of experience in superficial US and 6 years in CEUS). If disagreements occurred, a third senior radiologist (Y.J.C, with 15 years of experience) made the final decision. Cervical lymphoma suspicion on US was based on^14^: (a) absence of lymphatic hilum, (b) non-hilar blood flow.

### CEUS characteristics

#### Vascular phase

1. Enhancement patterns: Centripetal (from periphery to center), centrifugal (from center outward), or mixed.
2. Peak enhancement intensity: Compared to the adjacent muscle tissue at peak enhancement, the degree of enhancement is classified as high, equal, or low.
3. Enhancement uniformity: homogeneous (complete perfusion without defects) or heterogeneous (focal perfusion defects or mixed enhancement).

#### Postvascular phase

**Fig. 2:**
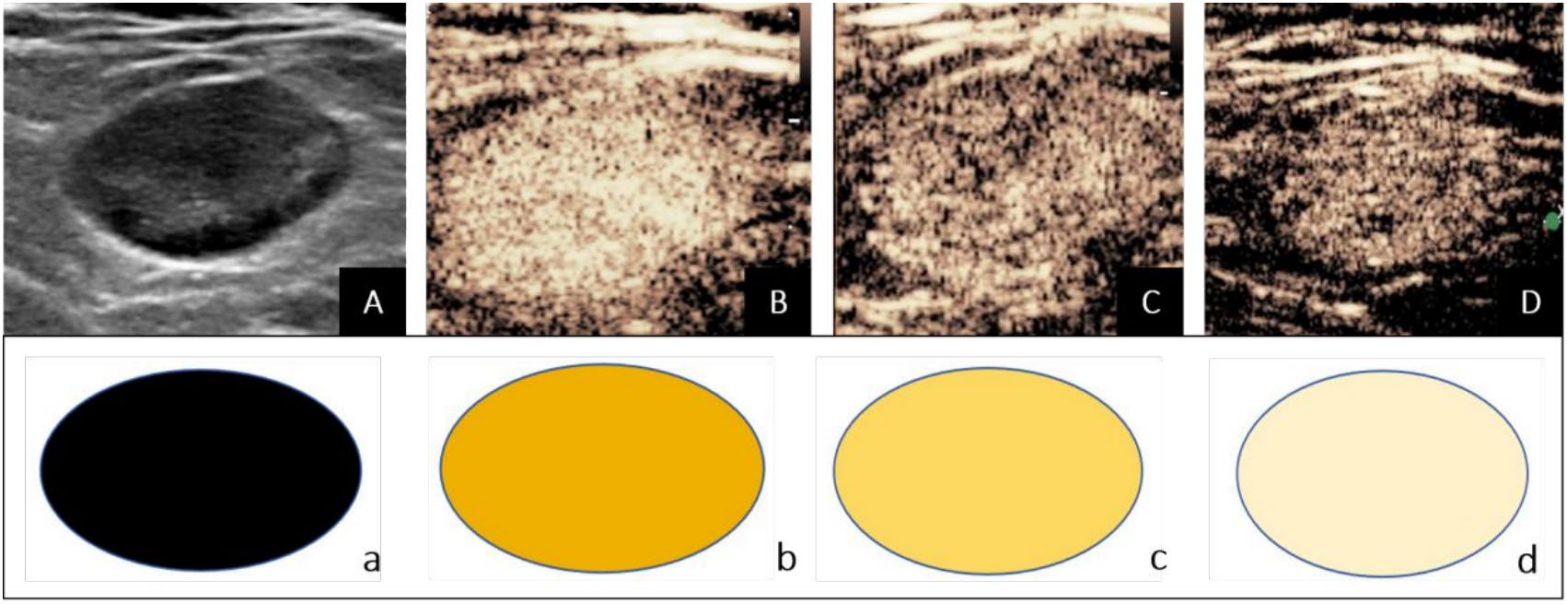
Images from a 60-year-old male patient with an enlarged lymph node in the right cervical level IV region, histologically confirmed as reactive hyperplasia. (**A**) Ultrasound image shows an 18 mm × 10 mm suspicious cervical lymph node (arrow), with a plump shape, uneven cortical thickening, and a narrowed, displaced hilum. The hilum shows a detectable blood flow signal. (**B**) Contrast-enhanced ultrasound (CEUS) in the early enhancement phase shows uniform high enhancement. (**C**) In the late enhancement phase, uniform slightly higher enhancement is observed. (**D**) In the postvascular phase, there is no significant regression, and the lymph node shows sustained uniform enhancement. This persistent homogeneous enhancement throughout the node, without perfusion defects or focal hypoenhancement, meets the criteria we define as the "sunburst sign." This sign is characterized by complete, uniform enhancement ≥ 10 min post-injection, indicating preserved macrophage function and likely benignity. Panels a, b, c, and d correspond to schematic diagrams: (a) two-dimensional image, (b) early enhancement, (c) late enhancement, (d) postvascular phase.

**Fig. 3:**
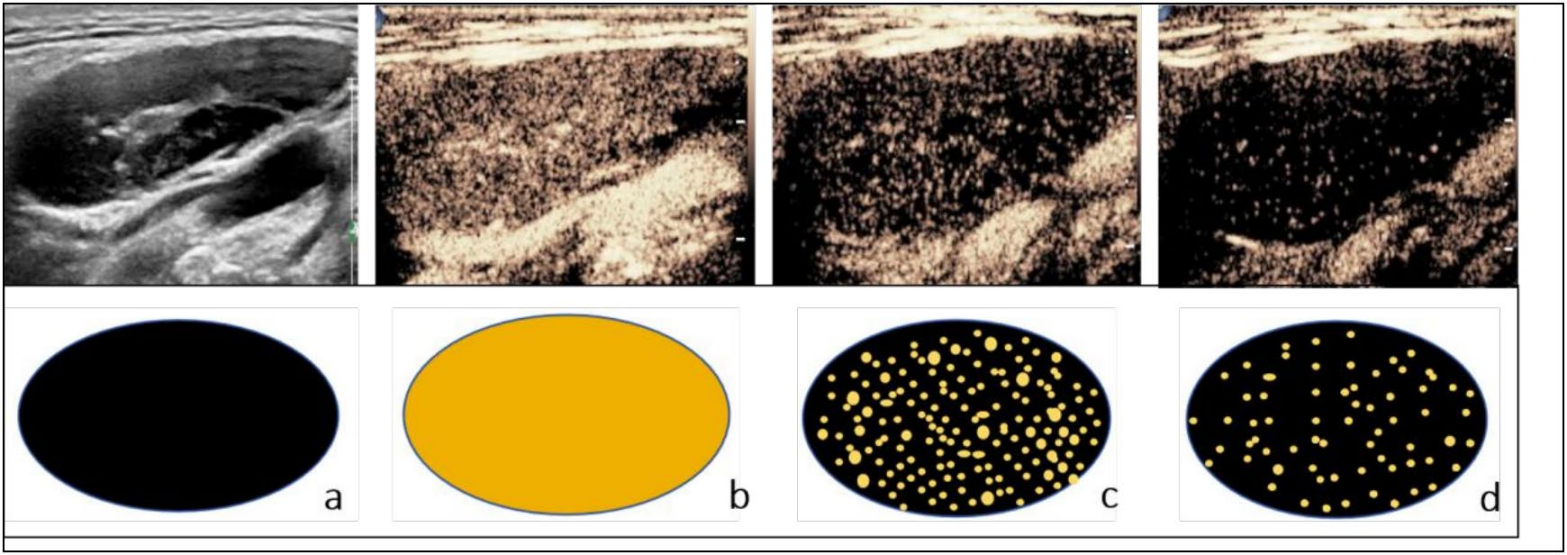
A 71-year-old female patient histologically confirmed with follicular lymphoma. (**A**) Ultrasound image shows a suspicious 37 mm × 12 mm cervical lymph node (arrow), elliptical in shape with uniform cortical thickening. The hilum and hilar blood flow signal are visible, resembling a benign lymph node. (**B**) Contrast-enhanced ultrasound (CEUS) in the early enhancement phase shows uniform high enhancement. (**C**) In the late enhancement phase, rapid regression occurs, with uneven enhancement. (**D**) In the postvascular phase, the lymph node exhibits large perfusion defects, with residual enhancement showing a scattered, spotty distribution. This enhancement pattern is defined as the “starfield sign”—marked by heterogeneous hypoenhancement with multiple discrete, punctate enhancing foci throughout the node in the postvascular phase (10–30 min). This pattern reflects disrupted macrophage distribution and suggests malignancy. Panels a, b, c, and d correspond to schematic diagrams: (a) two-dimensional image, (b) early enhancement, (c) late enhancement, (d) postvascular phase.

Enhancement patterns were categorized as follows:

1. Sunburst Sign: Uniform enhancement of the LN (Fig. 2).
2. Starfield Sign: Uneven low enhancement with scattered residual enhancement spots (Fig. 3).
3. Black Hole Sign: Complete absence of enhancement.

### Diagnostic evaluation

The diagnostic performance of US and CEUS was assessed using sensitivity, specificity, accuracy, positive predictive value (PPV), negative predictive value (NPV), and the area under the receiver operating characteristic curve (AUC), with histopathology serving as the reference standard. Benign lymph nodes were confirmed by histopathological and immunohistochemical analyses of surgical specimens (e.g., reactive hyperplasia, granulomatous inflammation) with no evidence of malignancy, complemented by clinical/imaging stability during follow-up (≥ 12 months).

### Statistical analysis

Statistical analyses were performed using SPSS (v20.0, IBM) and MedCalc (v22.001, MedCalc). Sample size calculation was conducted using PASS (v15.0, NCSS) with a one-sided McNemar test. Based on previous studies and our center’s experience, the accuracy of perflubutane CEUS in diagnosing cervical lymphoma increased from 50 to 80%^14^. With an alpha of 0.05 and a sample size of 23 pairs (46 subjects), the study achieved a power of 89.4% at a significance level of 0.05. Continuous variables were presented as mean ± standard deviation (SD) and analyzed using the t-test, while categorical variables were expressed as frequencies and percentages and analyzed using Pearson’s chi-squared test or Fisher’s exact test. Receiver operating characteristic (ROC) curve analysis was performed to determine the sensitivity, specificity, PPV, NPV, and accuracy of each diagnostic method. Logistic regression was used to evaluate the association between US, CEUS, and US combined with postvascular phase characteristics in diagnosing cervical lymphoma. The percentage of correct classifications, AUC, and 95% confidence interval (CI) were calculated for each method. The DeLong test was applied to compare differences in diagnostic performance among the models. A P-value of < 0.05 was considered statistically significant.

## Result

### Participant characteristics

Among the 47 patients included in the study, there were 25 males and 22 females. All cases underwent either surgery or CNB to obtain pathological samples. There were 23 cases of lymphoma and 24 cases of benign LNs. The mean age of patients with lymphoma was 60.39 ± 21.13 years, while the mean age of patients with benign LNs was 50.29 ± 18.76 years. The histological types of the 23 lymphoma cases were as follows: diffuse large B-cell lymphoma (n = 11), T-cell lymphoma (n = 6), follicular lymphoma (n = 2), small B-cell lymphoma (n = 2), marginal zone mucosa-associated lymphoid tissue lymphoma (n = 1), and classical Hodgkin lymphoma (n = 1). The demographic and lesion size data of the two groups are shown in Table 1. There were no statistically significant differences in age and gender between patients with lymphoma and those with benign LNs (*P* = 0.09 and 0.30, respectively). The follow-up period for benign LNs was more than 12 months(mean 13.7 ± 3.2 months). No changes in size or characteristics of these LNs were observed in follow-up US examinations.

**Table 1:** Basic characteristics of patients with lymph nodes.

Characteristic	Lymphoma	Benign LNs	*P*-value
Age(y)	60.39 ± 21.13	50.29 ± 18.76	0.09
Sex			
Male	14	11	0.30
Female	9	13	
Maximum diameter(cm)	2.28 ± 0.74	2.36 ± 0.93	0.04

Data are means ± standard deviation, LN: lymph node.

### Diagnostic agreement between the two radiologists

Among the 47 lesions, 42 (93.62%) had concordant results from both radiologists using conventional US, with 5 lesions (6.38%) showing discrepancies. Using CEUS, 44 lesions (95.74%) had concordant results, with 3 lesions (4.26%) showing discrepancies. The kappa values for the diagnostic results were 0.79 (95% CI 0.61, 0.97) for US and 0.87 (95% CI 0.73, 1.01) for CEUS. These kappa values, both above 0.75, indicate good diagnostic agreement between the two radiologists.

### Comparison of clinical and US indicators

Univariate analysis showed significant differences between lymphoma and benign LNs in terms of maximum lesion diameter (Table 1), enhancement direction, and postvascular phase (Table 2). Multivariate analysis was used to identify independent indicators of lymphoma. The results showed that the starfield sign was an independent indicator associated with lymphoma (Table 3). The AUC was 0.87 (95% CI 0.76–0.98), with a sensitivity of 91.30%, specificity of 83.33%, positive predictive value of 84.00%, and negative predictive value of 90.91%. The sunburst sign was an independent indicator associated with benign LNs. The AUC was 0.81 (95% CI 0.68–0.94), with a sensitivity of 70.83%, specificity of 91.30%, positive predictive value of 89.47%, and negative predictive value of 75.00%.

**Table 2:** Univariate analysis of US and CEUS characteristics.

Modality and feature	Description	Lymphoma	Benign LNs	*P*-value
US				
Shape	Round/oval	16	22	0.06
	Irregular	7	2	
Smooth edge	Yes	21	23	0.61
	No	2	1	
Echogenicity	Hypo-echoic	22	23	1.00
	Mixed-echoic	1	1	
Cystic degeneration	Yes	2	1	0.61
	No	21	23	
Homogeneous echo	Yes	12	19	0.06
	No	11	5	
Hilum	Yes	9	14	0.19
	No	14	10	
Portal blood flow	Yes	9	16	0.06
	No	14	8	
CEUS				
Vascular phase				
Perfusion pattern	Centripetal	17	11	0.02
	Centrifugal	2	11	
	Diffuse	4	2	
Enhancement intensity	Hyperenhancement	15	20	0.09
	Isoenhancement	1	4	
	Hypoenhancement	7	0	
Homogeneity	Homogeneous	22	23	0.71
	Heterogeneous	1	1	
Postvascular phase	Sunburst sign	2	17	< 0.01
	Starfield sign	21	4	
	Black hole sign	0	3	

US: ultrasound, CEUS: contrast-enhanced ultrasound, LN = lymph node, Categorical variables were analyzed using Pearson’s chi-squared test and Fisher’s exact test.

**Table 3:** Multivariable analysis of US and CEUS.

Description	B	SE	OR (95%CIs)	*P*-value
Maximum diameter	− 0.30	0.55	0.74 (0.26–2.16)	0.58
Centripetal	− 1.59	1.16	0.20 (0.02–1.99)	0.17
Diffuse	− 1.97	1.57	0.14 (0.01–3.04)	0.21
Starfield sign	− 3.71	0.96	0.03 (0.00–0.16)	< 0.01

B: regression coefficient, SE: standard error; OR (95% CI): odds ratio (95% confidence interval), US: ultrasound, CEUS: contrast-enhanced ultrasound.

### Diagnostic efficacy of US and CEUS in cervical LNs

*Overall performance of US:* Of the 32 LNs diagnosed as lymphoma by US, 15 cases (46.88%) were confirmed by pathology. The sensitivity, specificity, accuracy, positive predictive value, and negative predictive value of US for cervical lymphoma were 46.88% (15/32 LNs, 95% CI 0.30%, 0.65%), 46.67% (7/15 LNs, 95% CI 0.22%, 0.73%), 46.81% (22/47 LNs, 95% CI 32.55%, 61.07%), 65.22% (15/23 LNs, 95% CI 42.82%, 82.81%), and 29.17% (7/24 LNs, 95% CI 13.44%, 51.25%), respectively.

*Overall performance of CEUS:* Of the 23 LNs diagnosed as lymphoma by CEUS, 21 cases (91.30%) were confirmed by pathology, while 2 cases (8.70%) were ultimately diagnosed as benign LNs. Multivariate analysis showed that postvascular phase starfield sign in CEUS is a specific feature of lymphoma (Fig. 3). When the maximum lesion diameter is greater than 3 cm, the sensitivity of starfield sign in the postvascular phase can reach 100% (Table 4). The sensitivity, specificity, accuracy, positive predictive value, and negative predictive value of CEUS for cervical lymphoma were 87.50% (21 of 24 LNs; 95% CI 66.54%, 96.71%), 91.30% (21 of 23 LNs; 95% CI 70.49%, 98.48%), 89.36% (42 of 47 LNs; 95% CI 80.54%, 98.18%), 91.30% (21 of 23 LNs; 95% CI 70.49%, 98.48%), and 87.50% (21 of 24 LNs; 95% CI 66.54%, 96.71%), respectively. The diagnostic performance of US and LCEUS is shown in Table 5.

**Table 4:** Performance of Starfield Sign based on LNs size.

LNs size	N	Starfield sign (n)	Correct proportion
> 3 cm (n = 18)			
Lymphoma	10	10	100.00%
Benign LNs	7	2	28.57%
≤ 3 cm (n = 29)			
Lymphoma	13	10	76.92%
Benign LNs	17	2	11.76%

LN = lymph node.

**Table 5:** Diagnostic performance of US, CEUS, and US combined with postvascular phase.

Model and actual groups	Predicted groups of neck LNs		Correct proportion	AUC (95%CIs)	Youden Index J
	Lymphoma	Benign LNs			
US					
Lymphoma (n = 23)	15	8	15/23 (65.2)	0.68 (0.53–0.84)	0.36
Benign LNs (n = 24)	17	7	7/24 (29.2)	–	–
Correct proportion	–	–	22/47 (46.8)	–	–
CEUS					
Lymphoma (n = 23)	21	2	21/23 (91.3)	0.89 (0.79–1.00)	0.79
Benign LNs (n = 24)	3	21	21/24 (87.5)	–	–
Correct proportion	–	–	42/47 (89.4)	–	–
US combined with postvascular phase					
Lymphoma (n = 23)	22	1	22/23 (95.7)	0.92 (0.82–1.00)	0.83
Benign LNs (n = 24)	3	21	21/24 (87.5)	–	–
Benign LNs	–	–	43/47 (91.5)	–	–

A multivariable logistic regression model was used to evaluate diagnostic performance. Both the actual group and the predicted group included 47 cases of suspicious cervical lymphomas. The actual group comprised histologically confirmed benign LNs and lymphomas, while the predicted group comprised benign LNs and lymphomas classified by the diagnostic model. LN: Lymph node, AUC: Area under the receiver operating characteristic curve, US: ultrasound, CEUS: Contrast-enhanced ultrasound. Data represent the number of LNs, with percentages in parentheses.

**Fig. 4:**
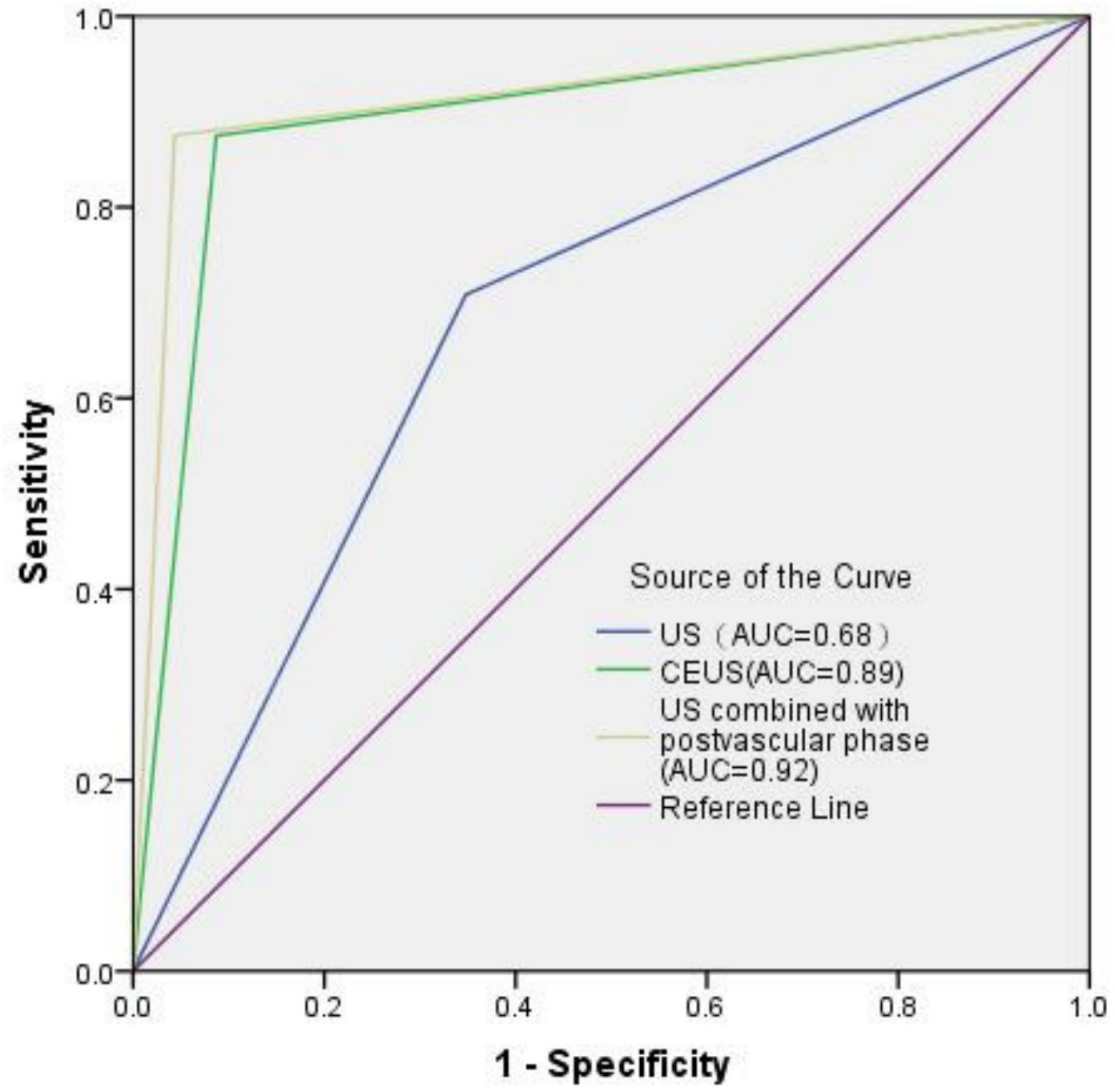
The receiver operating characteristic (ROC) curves show the diagnostic performance of US, CEUS, and the combination of US with the postvascular phase features, along with the area under the curve (AUC) for each. US: ultrasound, CEUS: contrast-enhanced ultrasound.

The AUC for the combination of the postvascular phase and US feathe postvascular phasetures was 0.92 (95% CI 0.82–1.00), significantly higher than that for US features alone (AUC, 0.68; 95% CI 0.53, 0.84; P < 0.05), as shown in Table 5 and Fig. 4.

## Discussion

Cervical lymphoma is often simply distinguished from benign LNs by determining the presence of the hilum. Lymphomas within the LNs grow from the inside out, and early lymphomas cause minimal damage to the small single arteriovenous structures of the hilum, which may present as gate-like blood flow signals. Therefore, it is difficult to differentiate from benign enlarged LNs. In this study, the hilum was present in 39.13% of lymphomas (9 of 23 cases) and in 58.33% of benign LNs (14 of 24 cases). Comparing the two groups under conventional US, no significant differences were found in other indicators except for the presence of the hilum. Lymphomas rarely undergo necrosis^25^ and most show homogeneous high enhancement in the arterial phase on CEUS^26^, similar to benign enlarged LNs. In this study, both lymphomas and benign LNs mostly exhibited homogeneous high enhancement (15 of 23 lymphomas and 20 of 24 benign LNs). Therefore, CEUS using sulfur hexafluoride as a contrast agent has limited diagnostic value for evaluating cervical lymphoma^15^. European guidelines also discuss the limitations of CEUS in lymphoma and do not recommend its routine use for differentiating lymphoma from benign LNs, recommending CEUS only in special circumstances^27^. In our clinical practice, we found that CEUS using Sonazoid (perfluorobutane microbubble contrast agent) has its own advantages in lymphoma. Perfluorobutane can be phagocytosed by macrophages, causing the highlighted areas to appear, while areas without macrophages do not show enhancement. This prospective study found that CEUS using perfluorobutane as a contrast agent showed good diagnostic performance for suspected cervical lymphoma. The AUC for CEUS was 0.89. The AUC for the combination of the postvascular phase and US features was 0.92, significantly higher than the AUC for US features alone (*P* < 0.05). Notably, the postvascular phase starfield sign demonstrated strong diagnostic performance. The combination of US with the postvascular phase features improved the accurate diagnosis of 7 cervical lymphomas and 14 benign LNs.

Normal LNs contain macrophages in both the cortex and medulla^20,21^. When foreign substances (such as perfluorobutane) enter the LNs through the bloodstream^28,29^, they can be phagocytosed by macrophages. Therefore, benign LNs exhibit uniform enhancement during the postvascular phase^30^, which we describe as the “sunburst sign”(Fig. 2). The sunburst sign is an independent indicator associated with benign LNs. Its AUC is 0.81, with a sensitivity of 70.83%, specificity of 91.30%, positive predictive value of 89.47%, and negative predictive value of 75.00%. Therefore, LNs exhibiting the sunburst sign in the postvascular phase are likely benign and may be exempt from biopsy. In contrast, lymphoma cells can invade and destroy the normal structure of LNs, leading to a significant reduction in macrophages and less phagocytosis of microbubbles. Therefore, in the postvascular phase, lymphomas show scattered punctate heterogeneous hypoechoic enhancement within the LNs, which we describe as the “starfield sign”(Fig. 3). Its sensitivity is 91.30%, specificity is 83.33%, positive predictive value is 84.00%, negative predictive value is 90.91%, AUC is 0.87, and diagnostic accuracy is 89% (23 of 21 LNs). There was no difference in the AUC between the combination of CEUS and postvascular phase and US features (*P* > 0.05). We observed that in lesions larger than 3 cm (21 out of 23 cases), uneven enhancement in the postvascular phase had a sensitivity of 100% for lymphoma, indicating strong diagnostic performance. Based on these results, we believe that CEUS using perfluorobutane as a contrast agent is a promising tool for differentiating suspected cervical lymphomas.

Notably, 3 cases of benign LNs showed no enhancement in the postvascular phase (black hole sign). The final pathological results for these 3 cases were non-tuberculous granulomatous lymphadenitis (2 cases) and necrotizing lymphadenitis (1 case). The possible reason for no enhancement in the postvascular phase is the presence of excessive fibrous tissue within the lesion and a lack of phagocytes, preventing contrast agent visualization in the lesion. This phenomenon was not observed in the postvascular phase of lymphomas. Therefore, we hypothesize that no enhancement in the postvascular phase may be seen in specific types of inflammatory lesions or other types of malignant tumors, which requires further research to confirm. Additionally, we observed that 4 cases of benign LNs showed heterogeneous hypoechoic enhancement (starfield sign) in the postvascular phase. This may be related to the formation of new fibrous tissue after the destruction of the normal structure of the LNs, leading to a reduction in phagocytes.

In the univariate analysis, the maximum tumor diameter on conventional ultrasound showed a statistically significant difference between lymphomas and benign LNs (*P* < 0.01). The maximum tumor diameter in lymphomas is larger than that in benign LNs, likely because lymphomas are malignant tumors with uncontrolled clonal proliferation, resulting in larger size. In contrast, most benign enlarged LNs are due to reactive hyperplasia, which is regulated growth, resulting in relatively smaller size. This study also found that the direction of enhancement on CEUS showed a statistically significant difference between the two groups (P = 0.02), with lymphomas more often exhibiting centripetal enhancement (17 of 23 cases). While previous studies have reported that only approximately half of lymphomas show centripetal enhancement (e.g., 58.73%), this difference may not solely be explained by selection bias. Other contributing factors may include differences in the contrast agent used (perfluorobutane vs. sulfur hexafluoride), tumor subtype composition, vascular remodeling patterns, and operator technique. Further comparative studies across multiple centers using standardized protocols are needed to clarify the variability in enhancement patterns observed in lymphoma. If the hilar vessels are destroyed and tumor neovascularization predominates, it can result in centripetal enhancement. If hilar vessels predominate, centrifugal enhancement may occur, a phenomenon also reported in the literature^31^.

Our study has several limitations. First, it is a single-center study with a small sample size, which may limit statistical power and generalizability. Second, selection bias may exist, as only patients already scheduled for biopsy were included, excluding borderline cases commonly seen in clinical practice. Third, metastatic and tuberculous lymph nodes were not included as comparison groups, limiting evaluation of the starfield sign’s specificity across diverse pathologies. Fourth, CEUS was performed by a single radiologist involved in study design, which may introduce observer bias. Fifth, some benign lymph nodes were classified based on core needle biopsy and short-term follow-up (mean 13.7 months), which may not fully exclude indolent malignancies. Sixth, the study relied on subjective visual signs without quantitative CEUS parameters, which may affect reproducibility. Finally, the novel terms “starfield sign” and “sunburst sign” lack standardized definitions, which may limit cross-institutional application and require further validation.

## Conclusion

In conclusion, CEUS using perfluorobutane as a contrast agent can effectively differentiate cervical lymphomas from benign LNs, especially with the starfield sign showing good diagnostic performance. The sunburst sign indicates a high likelihood of benignity, potentially avoiding unnecessary biopsy. Further large-scale, multicenter studies are needed to validate these findings.

## Data Availability

The datasets used and/or analysed during the current study available from the corresponding author on reasonable request.

## Abbreviations

LN: Lymph node
CEUS: Contrast-enhanced ultrasound
US: Ultrasound
AUC: Area under the receiver operating characteristic curve
CNB: Core needle biopsy
ROC: Receiver operating characteristic

## References

[1] Cooper, J. S. et al. National Cancer Database report on cancer of the head and neck: 10-year update. *Head Neck.* **31**(6), 748–758 (2009). 19189340 10.1002/hed.21022

[2] Sohani, A. R. & Hasserjian, R. P. Diagnosis of Burkitt lymphoma and related highgrad B-cell neoplasms. *Surg. Pathol. Clin.* **3**(4), 1035–1059 (2010). 26839298 10.1016/j.path.2010.09.010

[3] Toader, C. et al. Tonsillar lymphoma masquerading as obstructive sleep apnea - pediatric case report. *Rom. J. Morphol. Embryol.* **57**(2 Suppl), 885–891 (2016). 27833988

[4] Perry, A. M. et al. Non-hodgkin lymphoma in the far east: review of 730 cases from the international non-hodgkin lymphoma classification project. *Ann. Hematol.* **95**, 245–251 (2016). 26537613 10.1007/s00277-015-2543-4

[5] Rubaltelli, L. et al. Evaluation of lymph node perfusion using continuous mode harmonic ultrasonography with a second-generation contrast agent. *J. Ultrasound Med.* **23**, 829–836 (2004). 15244307 10.7863/jum.2004.23.6.829

[6] Ma, X. et al. Application of contrast-enhanced ultrasound (CEUS) in lymphomatous lymph nodes: a comparison between PET/CT and contrast-enhanced CT. *Contrast Media Mol. Imaging.* **2019**, 5709698 (2019). 30809108 10.1155/2019/5709698 PMC6364116

[7] Jiang, W. et al. Value of contrast-enhanced ultrasound and PET/CT in assessment of extramedullary lymphoma. *Eur. J. Radiol.* **99**, 88–93 (2018). 29362156 10.1016/j.ejrad.2017.12.001

[8] Esen, G. Ultrasound of superficial lymph nodes. *Eur. J. Radiol.* **3**, 345–359 (2006). 10.1016/j.ejrad.2005.12.039 16480846

[9] Ma, X. et al. Application of contrast-enhanced ultrasound (CEUS) in lymphomatous 304 lymph nodes: a comparison between PET/CT and contrast-enhanced CT. *Contrast Media Mol. Imaging.* **2019**, 5709698 (2019). 30809108 10.1155/2019/5709698 PMC6364116

[10] Jiang, W. et al. Value of contrast-enhanced ultrasound and PET/CT in assessment 307 of extramedullary lymphoma. *Eur. J. Radiol.* **99**, 88–93 (2018). 29362156 10.1016/j.ejrad.2017.12.001

[11] Rubaltelli, L. et al. Evaluation of lymph node perfusion using continuous mode harmonic ultrasonography with a second-generation contrast agent. *J. Ultrasound Med.* **23**(6), 829–836 (2004). 15244307 10.7863/jum.2004.23.6.829

[12] Mei, M., Ye, L., Quan, J. & Huang, P. Contrast-enhanced ultrasound for the differential diagnosis between benign and metastatic superficial lymph nodes: a meta-analysis. *Cancer Manag. Res.* **10**, 4987–4997 (2018). 30464599 10.2147/CMAR.S174751 PMC6208530

[13] Xiao, L. et al. Contrast-enhanced us with perfluorobutane to diagnose small lateral cervical lymph node metastases of papillary thyroid carcinoma. *Radiology* **307**(4), e221465 (2023). 37014242 10.1148/radiol.221465

[14] Liu, N. et al. A combination of ultrasound and contrast-enhanced ultrasound improves diagnostic accuracy for the differentiation of cervical tuberculous lymphadenitis from primary lymphoma. *Clin. Hemorheol. Microcirc.* **85**(3), 261–275 (2023). 37599529 10.3233/CH-231876

[15] Nie, J. et al. The value of CEUS in distinguishing cancerous lymph nodes from the primary lymphoma of the head and neck. *Front. Oncol.* **10**, 473 (2020). 32373513 10.3389/fonc.2020.00473 PMC7186353

[16] Watanabe, R., Matsumura, M., Chen, C. J., Kaneda, Y. & Fujimaki, M. Characterization of tumor imaging with microbubble-based ultrasound contrast agent, sonazoid, in rabbit liver. *Biol. Pharm. Bull.* **28**(6), 972–977 (2005). 15930729 10.1248/bpb.28.972

[17] Watanabe, R. et al. Gray-scale liver enhancement with Sonazoid (NC100100), a novel ultrasound contrast agent; detection of hepatic tumors in a rabbit model. *Biol. Pharm. Bull.* **26**(9), 1272–1277 (2003). 12951470 10.1248/bpb.26.1272

[18] Watanabe, R., Matsumura, M., Munemasa, T., Fujimaki, M. & Suematsu, M. Mechanism of hepatic parenchyma-specific contrast of microbubblebased contrast agent for ultrasonography: microscopic studies in rat liver. *Invest. Radiol.* **42**(9), 643–651 (2007). 17700280 10.1097/RLI.0b013e31805f2682

[19] Yanagisawa, K., Moriyasu, F., Miyahara, T., Yuki, M. & Iijima, H. Phagocytosis of ultrasound contrast agent microbubbles by Kupffer cells. *Ultrasound Med. Biol.* **33**(2), 318–325 (2007). 17207907 10.1016/j.ultrasmedbio.2006.08.008

[20] Bellomo, A., Gentek, R., Bajénoff, M. & Baratin, M. Lymph node macrophages: Scavengers, immune sentinels and trophic effectors. *Cell Immunol.* **330**, 168–174 (2018). 29397903 10.1016/j.cellimm.2018.01.010

[21] Dietrich, C. F. et al. Guidelines and good clinical practice recommendations for contrast enhanced ultrasound (CEUS) in the liver - update 2020 - WFUMB in cooperation with EFSUMB, AFSUMB, AIUM, and FLAUS. *Ultraschall. Med.* **41**(5), 562–585 (2020). 32707595 10.1055/a-1177-0530

[22] Goldberg, B. B. et al. Contrast-enhanced ultrasound imaging of sentinel lymph nodes after peritumoral administration of sonazoid in a melanoma tumor animal model. *J. Ultrasound Med.* **30**(4), 441–453 (2011). 21460143 10.7863/jum.2011.30.4.441

[23] Goldberg, B. B., Merton, D. A., Liu, J. B., Murphy, G. & Forsberg, F. Contrast-enhanced sonographic imaging of lymphatic channels and sentinel lymph nodes. *J. Ultrasound Med.* **24**(7), 953–965 (2005). 15972710 10.7863/jum.2005.24.7.953

[24] Piscaglia, F et al. The EFSUMB Guidelines and Recommendations on the Clinical Practice of Contrast Enhanced Ultrasound (CEUS): Update 2011 on non-hepatic applications. 10.1055/s-0031-1281676 21874631

[25] King, A. D., Lei, K. I. & Ahuja, A. T. MRI of neck nodes in non-Hodgkin’s lymphoma of the head and neck. *Br. J. Radiol.* **77**, 111–115 (2004). 15010382 10.1259/bjr/53555208

[26] Slaisova, R. et al. Contrast-enhanced ultrasonography compared to gray-scale and power Doppler in the diagnosis of peripheral lymphadenopathy. *Eur. J. Radiol.* **82**(4), 693–698 (2013). 23298797 10.1016/j.ejrad.2012.12.008

[27] Dietrich, C., Maddalena, M., Cui, X., Schreiber-Dietrich, D. & Ignee, A. Liver tumor characterization-review of the literature. *Ultraschall in der Medizin-Eur. J. Ultrasound* **33**(1), S11–S21 (2012). 10.1055/s-0032-1312897 22723026

[28] Kesler, C. T., Liao, S., Munn, L. L. & Padera, T. P. Lymphatic vessels in health and disease. *Wiley Interdiscip. Rev. Syst. Biol. Med.* **5**(1), 111–124 (2013). 23209022 10.1002/wsbm.1201 PMC3527689

[29] Gray, E. E. & Cyster, J. G. Lymph node macrophages. *J. Innate Immun.* **4**(5–6), 424–436 (2012). 22488251 10.1159/000337007 PMC3574571

[30] Zhang, Y. et al. Lymphatic contrast-enhanced US to improve the diagnosis of cervical lymph node metastasis from thyroid cancer. *Radiology* **307**(4), e221265 (2023). 37014243 10.1148/radiol.221265

[31] Yu, M. et al. Clinical application of contrast-enhanced ultrasonography in diagnosis of superficial lymphadenopathy. *J. Ultrasound Med.* **29**(5), 735–740 (2010). 20427785 10.7863/jum.2010.29.5.735

